# Electrophysiological monitoring of nutrient stress in *Oscillatoria* sp. cohorts: Toward an early-warning tool for harmful algal blooms

**DOI:** 10.1557/s43580-025-01486-3

**Published:** 2025-12-15

**Authors:** Damiano Duci, Raquel Amaral, David M. S. Silva, Francisco C. Cotta, Felipe L. Bacellar, Lee Bryant, Rupert G. Perkins, Paulo R. F. Rocha

**Affiliations:** 1 Department of Architecture & Civil Engineering, Centre for Climate Adaptation & Environment Research (CAER), University of Bath, Bath, BA2 7AY UK; 2 Bioelectronics & Bioenergy Research Lab, Centre for Functional Ecology-Science for People & the Planet, Associate Laboratory TERRA, Department of Life Sciences, University of Coimbra, 3000-456 Coimbra, Portugal; 3 School of Earth and Environmental Sciences, Cardiff University, Park Place, Cardiff, CF10 3AT UK

## Abstract

Cyanobacterial harmful algal blooms (HABs) are a growing challenge for freshwater management, traditionally addressed through phosphorus (P) limitation strategies. However, increasing evidence highlights nitrogen (N) availability as a key driver of bloom initiation and persistence. Here, we report extracellular voltage recordings from Oscillatoria cohorts exposed to four days of N starvation followed by ammonium (NH₄⁺) repletion using 5 mg L^−1^. Under N deprivation, electrical signalling developed progressively with a median of 1–2 events min^−1^ and amplitudes between 3-4 µV on day 1 and 2, following a marked increase in activity by days 3 and 4, with median spike rates of 5 events min^−1^ and amplitudes up to 17 µV. Following NH_4_^+^ repletion, signalling activity declined within 24 h, indicating rapid restoration of N balance. These results suggest that electrophysiological monitoring can serve as a real-time, non-invasive indicator of nutrient stress in Oscillatoria and provides a potential early warning tool for HAB onset and associated taste and odour (T&O) outbreaks in freshwater systems.

**Graphical abstract:**

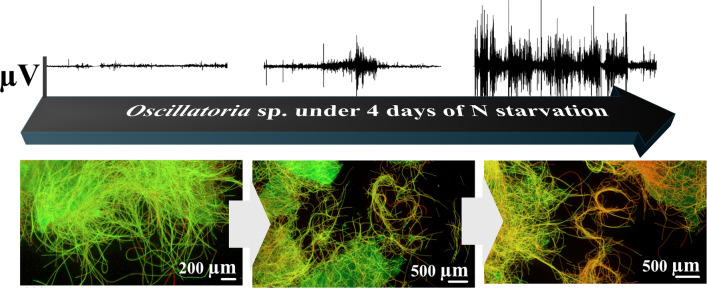

**Supplementary Information:**

The online version contains supplementary material available at 10.1557/s43580-025-01486-3.

## Introduction

Nutrient enrichment due to human-derived pollution discharge and climate change have increased the frequency, magnitude, and duration of cyanobacterial harmful algal blooms (HABs), which degrade water quality and impose major economic costs on water utilities [1]. In the USA alone, the annual cost of additional treatment is estimated at $1194 million [2]. While phosphorus (P) reduction has historically dominated management strategies, recent studies highlight that nitrogen (N) availability also plays a critical role in shaping bloom dynamics [1, 3]. Persistent low N conditions from denitrification, combined with internal P loading from sediments, create imbalances that favour cyanobacterial growth limited by N availability [4, 5]. Prolonged stratification, with earlier onset and later breakdown each year, further promotes cyanobacteria over competing phytoplankton, exacerbating HAB persistence [6, 7].

*Oscillatoria* is a filamentous cyanobacterium composed of serially arranged rectangular cells within cohesive filaments. It forms dense mats with coordinated gliding motility [8]. Importantly, *Oscillatoria* is a significant producer of taste and odour (T&O) compounds such as geosmin and 2-methylisoborneol (MIB), which burden water treatment even in the absence of cyanotoxins [4, 9]. Understanding its physiological responses to nutrient limitation is therefore critical for developing predictive monitoring strategies.

Beyond genetic and metabolic regulation, cyanobacteria also exhibit bioelectric activity. *Oscillatoria* cohorts have been shown to generate extracellular electrical fluctuations under dark stress, analogous to similar observations in diatom populations. Such findings suggest that *Oscillatoria* is an electrically active genus capable of stress-induced signalling at the cohort level [10, 11]. Bioelectricity is recorded using macroeletrode arrays (MEAs). Planar Au electrodes in close contact with microbial communities record the imbalance of ionic and redox charge fluxes occurring at the microbe electrode interface. Ions released or consumed during photosynthesis, respiration, and nutrient assimilation generate small potential differences within tens of microvolts, which can be recorded using a low-noise recording setup. These signals arise from the combined effect of transmembrane proton gradients, ion channels and redox reactions at the microbe–electrode interface that alter the local electrochemical potential of the surrounding electrolyte. Because the rates of the enumerated charge-transfer processes depend on the cell metabolic state, the variability in the recorded extracellular potential provides an electrical read-out, or proxy for metabolic activity. Under nutrient or ammonium stress, for example, changes in photosynthetic electron transport, nitrogen fixation or respiration are expected to modify the oxidation–reduction currents and ionic fluxes near the electrode, leading to characteristic oscillations, or fingerprints, in the measured potential. Thus, the extracellular potential recorded from microbial communities should function as a label-free real-time indicator of cellular metabolism and stress responses in cyanobacterial consortia. Bioelectric recordings therefore provide a complementary, non-invasive window into cyanobacterial physiology, with potential applications as early-warning indicators of HAB and T&O outbreaks.

Here, we investigate the electrophysiological response of *Oscillatoria* sp. under four days of nitrogen starvation followed by ammonium (NH_4_^+^) repletion at 5 mg L⁻^1^. We note that although nutrient concentrations in freshwater bodies are often monitored by water authorities at longer timescales (weeks to months), there remains a critical need for shorter timescales sensing where early metabolic changes could be preventive. We combine viability assays with extracellular voltage recordings and impedance spectroscopy to examine how nutrient stress influences cohort signalling. A characteristic electrical signature is found to develop under N starvation and to dissipate within seconds to minutes following NH_4_^+^ repletion. We propose that such electrical responses reflect coordinated metabolic and ion-channel dynamics within *Oscillatoria* sp. cohorts, and that these signatures could form the basis for real-time monitoring tools for nutrient stress and bloom prediction.

## Methods

### Viability assay of Oscillatoria sp. cohorts, under N starvation/replete conditions

A culture of *Oscillatoria* sp. was sourced from HAMBI culture collection (helsinky.fi/hambi), University of Helsinki, with the collection number UHCC 0332. To assess the tolerance of *Oscillatoria* sp. to nitrogen (N) starvation and its recovery following repletion, cultures were transferred from standard BG11 medium to BG11 lacking combined nitrogen, *e.g.* BG11–N (see Setup Day 0 in Supplementary Information). Cohorts were maintained for up to seven days in BG11–N, with viability assessed on days 1, 3, 5, and 7 using SYTO9/propidium iodide staining and confocal microscopy. Parallel cultures were supplemented with 5 mg L^−1^ NH_4_^+^ after 1, 3, or 5 days of starvation (Stock solution preparation of NH_4_Cl is detailed in Supplementary Information). Recovery was evaluated four days later, at days 5, 7, and 9, thus enabling comparison of starved and repleted samples against controls.

### Bioelectric activity under N starvation and repletion

Extracellular voltage recordings were performed using a custom glass microelectrode array (MEA) consisting of paired gold electrodes as previously detailed [12–14]. *Oscillatoria* sp. cohorts were seeded into wells and incubated under a 12 h light/dark cycle prior to recordings. To test the effect of N availability, wells were maintained either in BG11, BG11 –N, or BG11 –N supplemented with 5 mg L^−1^ NH₄⁺ a concentration chosen to mimic occasional combined sewer overflow events that can produce spikes around 5 mg/l of NH_4_^+^. Electrical activity was monitored over 6 days inside a Faraday´s cage. Dark conditions were maintained during all electrophysiological recordings. Impedance spectroscopy was performed prior to each recording to verify connection status (not shown). Signals were amplified with a transimpedance amplifier and analysed for spike frequency *e.g.,* events per minute, and magnitude, here represented in µV. Baseline measurements are shown in Fig. S1, in Supplementary Information.

**Fig. 1:**
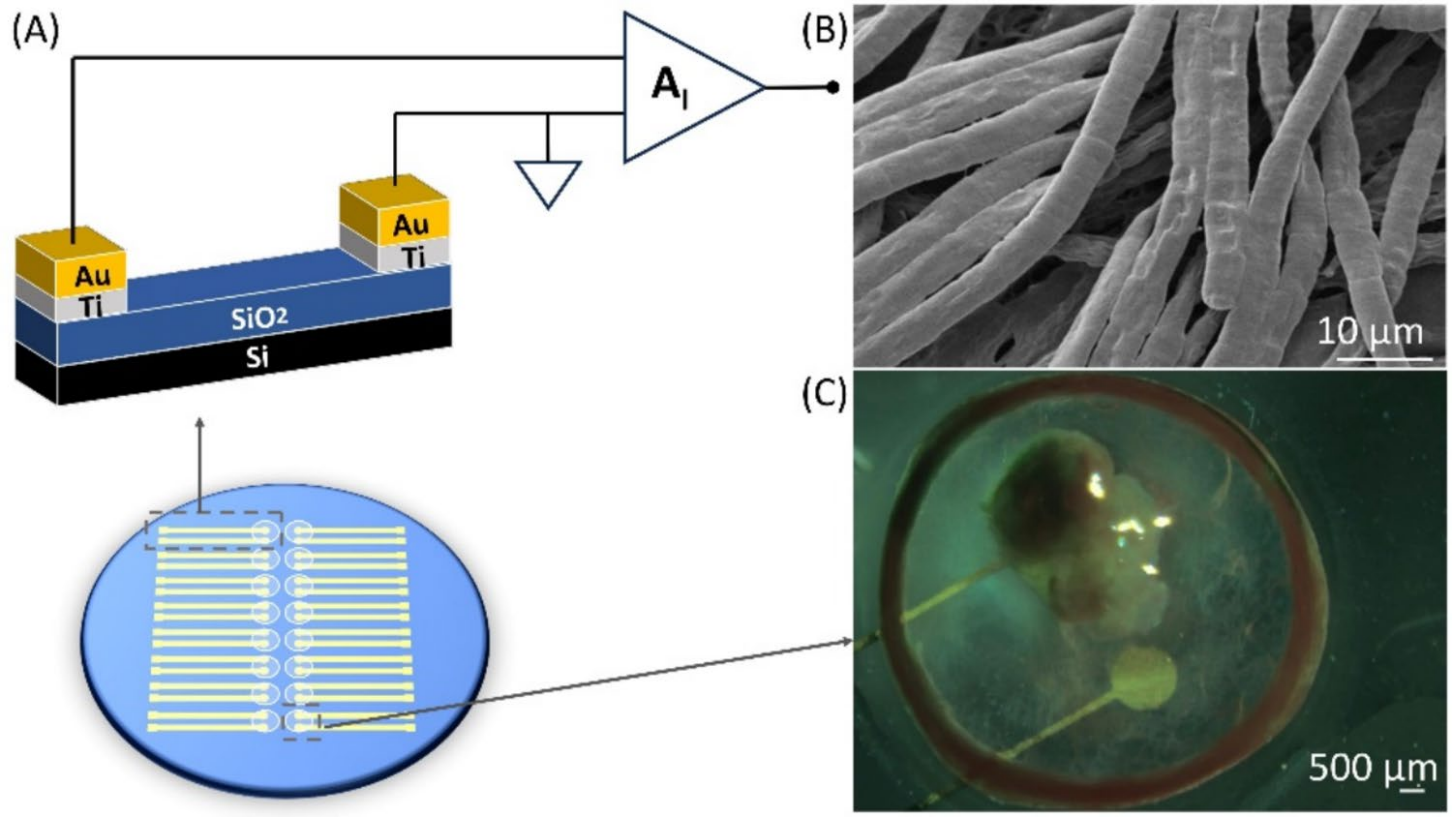
Electrode platform for *Oscillatoria* sp. extracellular recordings. **A** Schematic of the microelectrode device showing the layer stack and geometry comprising a glass substrate patterned with a pair of planar gold electrodes (measuring and counter area of 1.3 mm^2^ each) within a culture well. *Oscillatoria* sp. filaments settled and adhered over the exposed electrode surfaces during recordings. **B** Scanning electron micrograph of *Oscillatoria* sp. filaments adhering to the electrode surface (scale bar = 10 µm, magnification 2500x). **C** Photograph taken at day 7, of a gold electrode pair within the culture well, with filaments attached to the electrode area (scale bar 500 µm)

The custom electrode platform used for extracellular recordings is shown in Fig. 1A and has been explained in detail here [10–14]. In short, each well contains a pair of planar gold electrodes patterned on a glass substrate, and each electrode comprises an area of 1.3 mm^2^ each. During experiments, *Oscillatoria* sp. filaments adhere and form a biofilm over the exposed electrode surfaces, enabling stable, non-invasive voltage recordings under varying nitrogen regimes. Scanning electron microscopy (TESCAN VEGA 3 SBH Easy Probe SEM with a tungsten-heated cathode, images acquired with a working voltage of 5 kV using the secondary electron detector), in Fig. 1B, confirmed adhesion of *Oscillatoria* sp. filaments to the electrode surface (also observed in the micrograph of Fig. 1C). This configuration enabled stable, non-invasive recordings of *Oscillatoria* sp. bioelectric activity under varying nitrogen regimes.

## Results and discussion

### Viability assay under N availability starvation/replete conditions

**Fig. 2:**
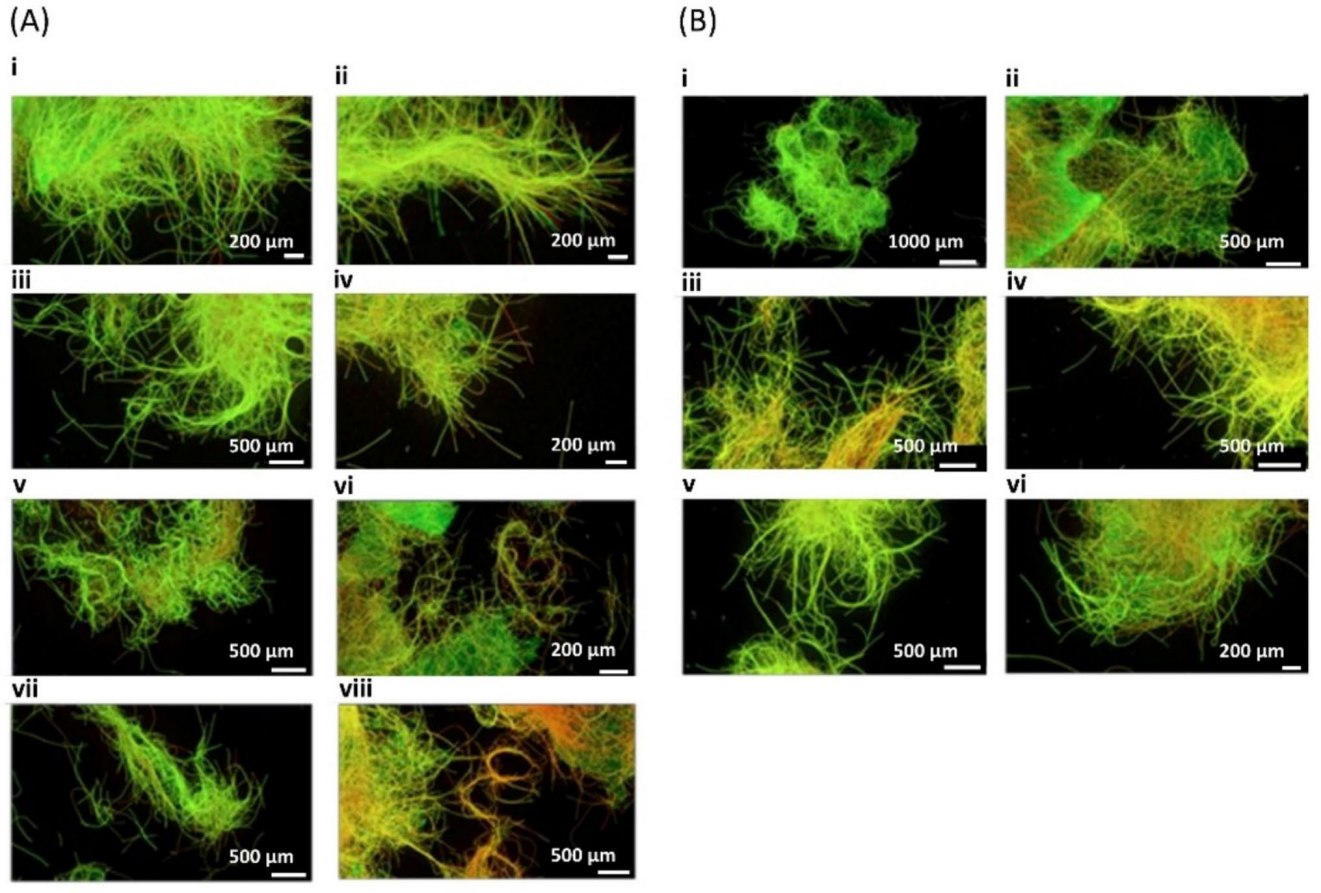
Live/Dead viability assay of *Oscillatoria* sp. cohorts during N starvation and recovery. **A** Panels (i), (iii), (v), (vi) show control cultures sampled at days 1, 3, 5, and 7, respectively. Panel ii-iv-vi-viii are the *Oscillatoria* sp. samples taken the same days grown in nitrogen deficient BG11 media, -N. By day 7, N-starved samples exhibited a stronger red coloration indicative of dead filaments, compared to controls and earlier time points. **B** Viability during recovery after NH_4_^+^supplementation. Panels (i), (iii), (v) show controls with no sign of stress at days 5, 7 and 9. No significant loss of viability or stress indicators were detected

*Oscillatoria* sp. cohorts remained viable for up to five days in BG11 -N, with no major differences compared to controls. By day 7, increased red fluorescence indicated loss of viability and onset of N stress chlorosis (Fig. 2A). Supplementation with NH₄⁺ with 5 mg L^−1^, after 1–5 days of starvation enabled full recovery within four days, confirming that transient N deprivation is reversible and compatible with subsequent electrophysiological monitoring.

### Bioelectric activity under N starvation/replete conditions

**Fig. 3:**
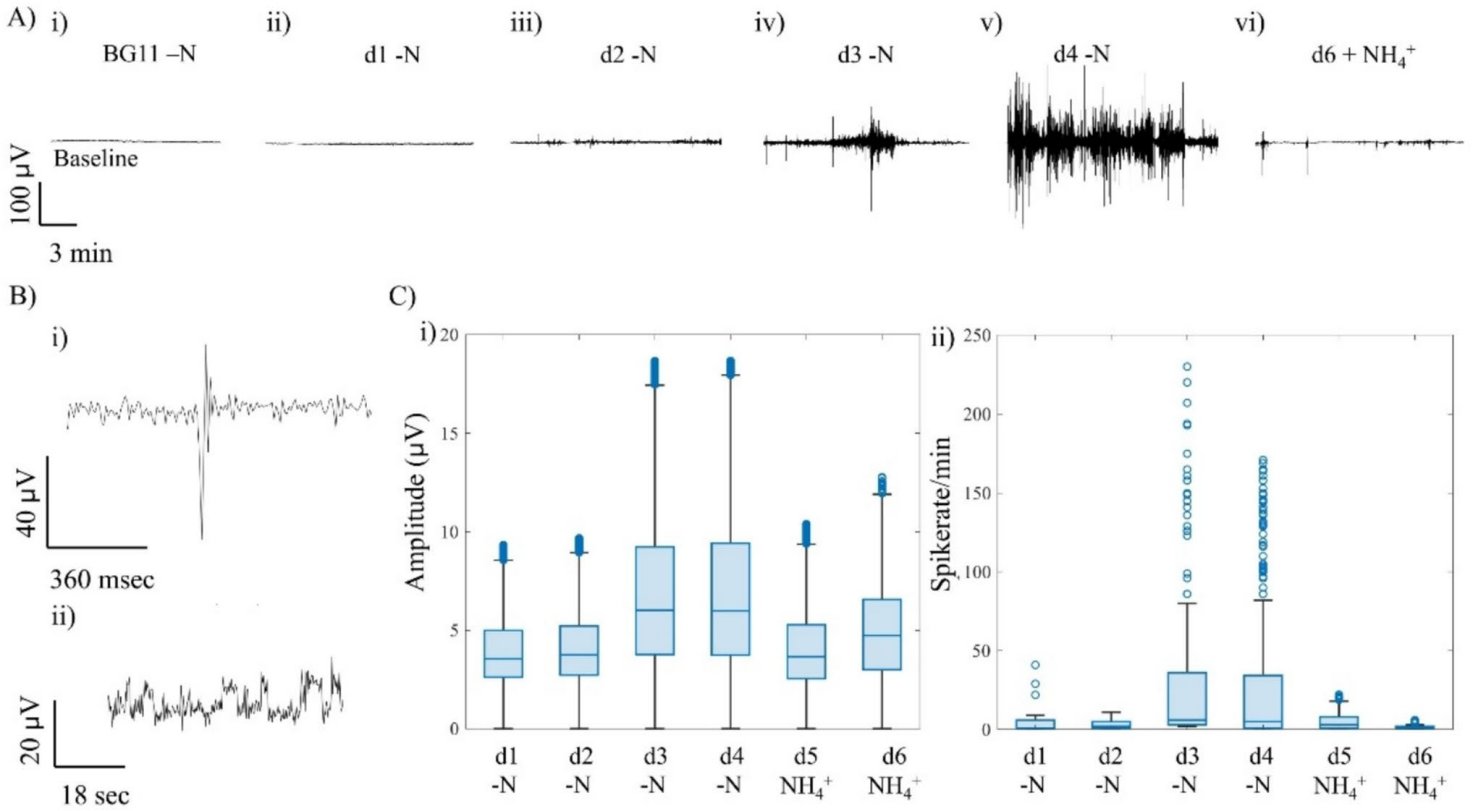
Electrical activity of *Oscillatoria* sp. cohorts under N starvation and NH_4_^+^ repletion.** A** Representative voltage traces showing increasing activity across days 1 to 4 of starvation and decline after NH_4_^+^ addition. **B** Two signal classes detected: (i) single high-amplitude spikes (milli-seconds scale) and (ii) slow random telegraph signal (RTS)-like fluctuations (seconds scale). **C** Boxplots of signal amplitude (i) and spike rate computed per minute (ii) across 6 days, where activity increased markedly on days 3 and 4 under starvation and declined rapidly following NH_4_^+^ repletion

On Day 1 to 4, the cell cohorts were starved from nitrogen with addition of BG11 -N, labelled as -N d1, -N d2, -N d3, and -N d4 (Fig. 3A). On the fifth day, 5 mg/L of NH₄⁺ is spiked into the well and activity is recorded for two more days labelled as NH_4_^+^ d1 and NH_4_^+^ d2. During starvation, a low spike rate is detected on day 1 and 2. Spiking activity was low with a median of 1–2 events min^−1^ and amplitudes between 3–4 µV (Table 1). As time elapsed, by days 3 and 4, the recorded electrical activity increased sharply, with median spike rates of 5 events min^−1^ and amplitudes up to 17 µV. In fact, two classes of events were observed, a fast spiking lasting milli-seconds and exhibiting high amplitude, and slower fluctuations, like a random telegraph signal (RTS) [11, 12], lasting seconds and exhibiting lower amplitudes (Fig. 3B).

**Table 1:** Median values detected in *Oscillatoria* sp

Day / condition	Nitrogen condition	Spike frequency (spikes/min)			Signal magnitude (µV)		
		Q2	IQR	Q4	Q2	IQR	Q4
–Nd1	N starvation (Day 1)	1	1–6	9	3.5	2.6–5.0	8.5
–Nd2	N starvation (Day 2)	2	1–5	11	3.7	2.7–5.2	9.0
–Nd3	N starvation (Day 3)	1	1–2	3	6.0	3.8–9.2	17.4
–Nd4	N starvation (Day 4)	5	1–32	82	6.0	3.7–9.4	17.0
NH_4_^+^d1	+ 5 mg/L NH₄⁺ addition (Day 5)	3	1–8	18	3.6	2.5–5.2	9.0
NH_4_^+^d2	+ 5 mg/L NH₄⁺ addition (Day 6)	1	1–2	3	4.7	3.0–6.5	12.0

Cohorts during the 6 days of experiments. *Q* quartile range, *IQR* interquartile range

After NH_4_^+^ repletion, the spike frequency and amplitudes promptly declined. Within 24 h, the electrical activity decreased toward baseline levels with a median of 3 events/min exhibiting about 4 µV in amplitude (Tab1). And after 48 h, the electrical signals recorded where comparable to that of controls, suggesting rapid restoration of N balance (Fig. 3C).

Nitrogen starvation induced a reproducible increase in the electrical activity of *Oscillatoria* sp., peaking after 3–4 days as viability markers indicated accumulated stress. This delayed response suggests an acclimatory phase rather than immediate shock. Under dark conditions, the signals likely reflect respiration-linked processes rather than photosynthetic activity. Metabolic reprogramming during N limitation alters redox balance and proton motive force, possibly leading to intermittent electrical bursts detectable at the cohort level.

We note the magnitude of the electrical bursts could also be modulated by biofilm architecture and cell density and further research is required to ascertain the mechanistic role of electrical excitability governing metabolic state. As *Oscillatoria* sp. establishes a confluent layer over the Au surface an increase in effective interfacial area and double-layer capacitance could in fact boost the low-frequency signal amplitude [11] and the growing series resistance due to the evolving exopolysaccharide (EPS) matrix thickness could elevate impedance and, in this way, attenuate higher frequency components. The emergence of long-lasting RTS-like signals implies coordinated stress responses, potentially mediated by ion channels and Ca^2^⁺ dynamics. Prior studies show that Ca^2^⁺ transients regulate membrane excitability and gene expression in cyanobacteria [15–18, 20]. Here, they may synchronize bursts within and between adjacent filaments, amplifying cohort-level electrogenic signatures [10, 11, 20]. While mechanistic details remain speculative, the observed activity aligns with known pathways of N stress adaptation, including phycobilisome dismantling, ribosome hibernation, and glycogen remobilization. [17–19].

NH_4_^+^ repletion rapidly suppressed bioelectric activity as viability recovered, consistent with assimilation via the GS-GOGAT pathway and restoration of C/N homeostasis. The timescale of recovery where the spike activity reduced within 24 h and arrived near baseline by 48 h, matches the biochemical kinetics of NH_4_^+^ assimilation and subsequent re-equilibration of cohort organization [17, 19].

Together, these findings demonstrate that *Oscillatoria* sp. exhibits distinct electrical signatures of N starvation and recovery. Such non-invasive electrophysiological monitoring could complement chemical and genetic assays, providing real-time indicators of nutrient stress relevant to HAB prediction. Previous electrophysiological studies on photosynthetic microorganisms have been mostly focusing on light-driven electron transport or extracellular electron transfer for bioelectrochemical energy conversion [21]. In contrast, to the best of our knowledge, no studies have addressed how nutrient stress modulates extracellular electrical signals in microalgae or diatoms, with the exception of our own recent perspective paper [22]. The present work shows that variations in nitrogen availability produce changes in the extracellular potentials recorded from *Oscillatoria* sp. biofilms, revealing a direct electrical signature of metabolic adaptation. This strongly suggests a new use for electrophysiology as a label-free probe for nutrient-related metabolic dynamics in photosynthetic microorganisms. The ability to detect early stress responses in T&O producing species positions this approach as a potential tool for water utilities seeking pre-emptive management strategies.

We note cyanotoxins or volatile metabolites was beyond the scope of this electrophysiological study. Yet, future work will seek to combine real-time electrical monitoring with chemical toxin and T&O quantification to evaluate whether the electrophysiological signatures identified here can serve as early indicators of harmful metabolite production.

## Conclusions

*Oscillatoria sp.* cohorts display distinct electrophysiological responses under nitrogen limitation and rapid suppression of activity following NH_4_^+^ repletion. Starvation for three to four days produced marked increases in spike frequency and amplitude, while NH_4_^+^ addition restored baseline activity within 48 h. These findings confirm that extracellular voltage recordings provide a non-invasive means of tracking nutrient stress and recovery in cyanobacterial populations. By linking nutrient availability to measurable electrogenic responses, this approach offers new opportunities for studying microbial physiology and developing real-time monitoring tools in aquatic systems.

## Supplementary Information

Below is the link to the electronic supplementary material.

Supplementary file1 (DOCX 452 KB)

## Data Availability

Supplementary data are available upon request to the authors.
