# Supplementary material for: Electrophysiological monitoring of nutrient stress in Oscillatoria sp. cohorts: Toward an early-warning tool for harmful algal blooms
Source: MRS Adv. 2025 Dec 15;10(23):2712–8. doi: 10.1557/s43580-025-01486-3 (PMC12756194; doi:10.1557/s43580-025-01486-3)
Supplement: Supplementary file 1 — Supplementary file1 (DOCX 452 KB) [file 43580_2025_1486_MOESM1_ESM.docx]

Supplementary information

Setup Day 0

*The cultures were grown in BG11 with pH 7.5 incubated in a growth chamber (Aralab, FC S600PLH) with a temperature of 18°C, photoperiod of 12-h:12-h light:dark, and a light intensity of 30 μmol/m2/s provided by cool white daylight fluorescent lamps. Oscillatoria* sp. culture is placed into a petri dish, half of the biomass is removed into BG11 deprived of N (BG11 -N) media and the other half in regular media. *Oscillatoria* sp. filaments are separated into an homogeneous culture to allow consistent sampling. Then, 1 mL of *Oscillatoria* sp. filament suspension was added in each well of two 12 well plates. Then, and 2 ml of regular BG11 is added to the controls and 2 mL of BG11 -N was added to the samples to begin N starvation. To change the *Oscillatoria* sp. filaments from the BG11 toBG11 -N condition, the culture was washed three times with BG11 -N, then placed in a 50 mL falcon and centrifuged for 5 mins at 500 rpm.

Stock solution preparation NH_4_Cl

A 250 ml volumetric was prepared, 100 ml of deionized water was added, then 0.25 g of NH_4_^+^ chloride (NH_4_Cl) was added, and water was filled up to volume. Then the NH_4_Cl solution was filtered with a 60 ml Luer syringe with a 0.2 µm pore Whatman puradisk filter. The NH_4_Cl solution was stored at 4 °C. Molarity of the stock solution was 18.7 mM of NH₄⁺. The volume to add for each sample was calculated as to obtain 5 mg/l of NH₄⁺ (Table S1).

**Table S1** The volume of each NH₄⁺ repletion was calculated based on the final volume of the culture containers used in different experiments, so that the final concentration is 5 mg/l of NH₄⁺ in all samples.

| Experiment | Volume container | Spike  volume | Final concentration NH₄⁺ |
| --- | --- | --- | --- |
| Viability assay | 3 ml | 57.3 µL | 5 mg/L |
| SPC monitor | 2 ml | 38.2 µL | 5 mg/L |
| Bioelectric activity | 0.5 ml | 9.5 µL | 5 mg/L |

Viability assay under N availability starvation/replete

The measurement method described is analogous in day 1, 3, 5, and 7 (Table 1). *Oscillatoria* sp. filaments were resuspended and 1 mL of *Oscillatoria* sp. culture was mixed with 3 µL SYTO9/propidium iodide (previously prepared 1:1 v/v), then covered in aluminium foil and kept in the dark for 15 minutes. *Oscillatoria* suspension was imaged (Axiocam 288 Zoom V.16, Zeiss). Samples Sd1_1-3 were spiked with 38.2 µL NH₄⁺ (5mg/L), and after 4 days the N effect of NH_4_^+^ replete was measured. Before spiking, 2 ml of BG11 was added to the controls and 2 ml of BG11 -N was added to the samples, to account for evaporation and maintaining the final calculated volume required for N replete concentration.

On day 3 the same staining procedure as day one is performed on controls Cd3_1-3 and samples Sd3_1-3. Then the samples are spiked with NH_4_^+^ to investigate the recovery of *Oscillatoria* sp. after 3 days of nitrogen starvation. Day 5 measurements as in day 1 and 3 were performed, and then the effect of NH_4_^+^ addition on day 1 was monitored i.e. 4 days from spiking. NH_4_^+^ samples were compared to controls n=3 from the same day. On day 7, the same procedure for day 1 was performed and the NH_4_^+^ samples spiked from day 3 were measured and compared to controls. Last measurements on day 9, only effect of NH_4_^+^ spike of samples on day 5 was measured.

Bioelectric activity under N starvation/replete conditions data

A control measurement for each media composition is measured with a sample size of n = 3 to ensure there are no baseline changes from recordings on gold electrodes with changing media. Figure 1 shows the baseline detection recording for all three media, BG11, BG11 without N, and BG11 with the addition of NH_4_^+^.


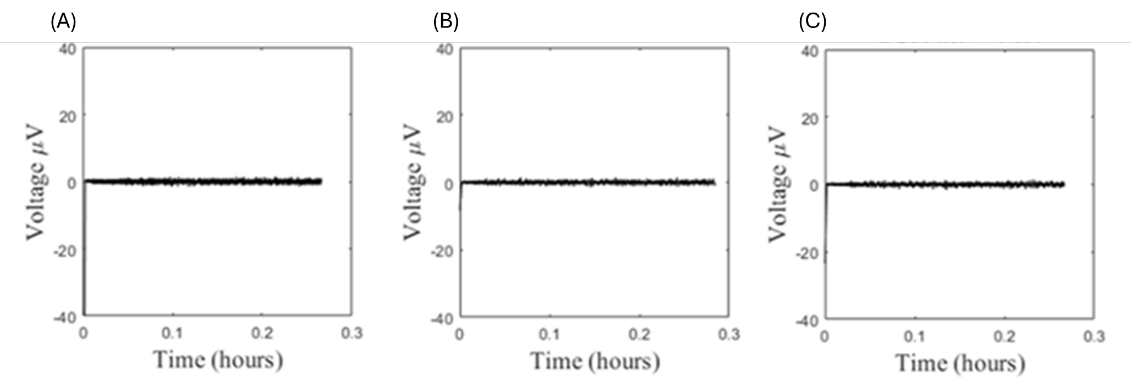


Fig. S1 Control measurements of different media compositions with n = 3 replicates. (A) Regular BG11 was monitored during 15 min. (B) BG11 -N is monitored for 15 min. (C) BG11+NH_4_^+^ is monitored for 15 min

On Day 1 to 4, the cell cohorts are starved from nitrogen with addition of BG11 noN, labelled as noNd1, noNd2, nond3, and noNday4 (Fig.3 C). On the fifth day, 5mg/L of NH_4_^+^ is spiked into the well and activity is recorded for two more days labelled as NH4d1 and NH4d2. Results show low spikerate/min activity on days noN1 with Q2 = 1 spike/min, IQR = 1 to 6 spikes/min, and Q4 = 9 spikes/min (Fig.3 C.ii). On day noN2 low spikerate is retained, with Q2= 2 spikes/min, IQR = 1 to 5 spikes/min, and Q4 = 11 spikes/min (Fig.3 C.ii). On noNd3 lower spikerate is recorded, with Q2 = 1 spike/min, IQR = 1 to 2 spikes/min, and a Q4= 3 spikes/min (Fig.3 C.ii). On day noN3 and 4 of recording, an increase in the spikerate is measured, with a Q2 = 5 spikes/min, IQR = 1 to 32 spikes/min, and Q4 = 82 spikes/min (Fig.3 C.ii). After NH_4_^+^ spiking on NH_4_^+^d1 a decrease in the spikerate occurs, with Q2 = 3 spikes/min, IQR = 1 to 8 spikes/min, and Q4 = 18 spikes/min. On NH_4_^+^d2 a further decrease of the signal spikerate occurs, with Q2 = 1 spike/min, IQR= 1 to 2 spikes/min, and Q4 = 3 spikes/min (Fig.3 C.ii). Concerning the signal magnitude ΔV measured in microvolts µV recordings results (Fig.3 C.i) show values on noNd1 of Q2= 3.5 µV, IQR = 2.6 to 5 µV, with a Q4 = 8.5 µV. On noNd2 the signal magnitude presents Q2 = 3.7 µV, IQR = 2.7 to 5.2 µV, and Q4 = 9 µV. On noNday3 an increase in the signal magnitude is recorded with Q2 = 6 µV, IQR = 3.8 µV to 9.2 µV, and a Q4 = 17.4 µV (Fig.3 C.i). On noNd4 the signal magnitude strength is retained, with Q2 = 6 µV, IQR = 3.7 µV to 9.4 µV, and Q4 = 17 µV. After NH_4_^+^ addition of 5 mg/L on day NH4d1, the signal magnitude decreases to values of Q2 = 3.6 µV, IQR = 2.5 µV to 5.2 µV, and Q4 = 9 µV. On day NH_4_^+^d2 a slight increase in the signals magnitude occurs, with Q2 = 4.7 µV, IQR = 3 µV to 6.5 µV, and Q4 = 12 µV (Fig.3 C.i).
